# The Influence of the Mind on the Diseases of the Body; or the Difference between Veterinary and Medical Science

**Published:** 1899-04

**Authors:** J. C. McKenzie

**Affiliations:** Rochester, N. Y.


### THE INFLUENCE OF THE MIND ON THE DISEASES OF THE BODY; OR THE DIFFERENCE BETWEEN VETERINARY AND MEDICAL SCIENCE.

By J. C. McKenzie, V.S.,
ROCHESTER, N. Y.

It will hardly be disputed, I think, that the veterinarian is entitled to a place among the members of the healing art. While doubtless medical practice and veterinary practice differ, nevertheless, they are branches of the same science. The physician has the advantage, however, that his skill is exercised upon beings endowed with intelligence, having the power of reason. This assists him greatly in his diagnoses, and his appeals to the intellectual faculties of his patient arouse valuable coöperation in combating and overcoming disease.

Now, while the physician, as a matter of necessity, familiarizes himself with all the varying pathological conditions, that one is best prepared to minister to the wants of the body, either in sickness or in health, who closely studies temporary states as well as permanent characteristics of the mind. The intimate connection between mind and body is illustrated in many ways, and is a subject of too general experience to require argument. Mind and body act and react upon each other, and the nerves, countless in number, all have their focus in the brain. Is the body ill, weak, or exhausted? To some extent the condition will be mirrored on the brain. Is the body stimulated or exhilarated? The brain shares its strengthened or quickened action. The influence of mind upon body is not less potent. Hope and joy quicken the circulation, brace the nerves, and give firmness and tension to the muscles. Grief and despondency have a relaxing tendency, weakening the limbs and deranging the various functions, more especially those of digestion and secretion.

That the influence of the mind upon a diseased body is often of great power for good or ill is a matter of record, and now seldom questioned. Eminent physicians have proved, beyond the shadow of a doubt, that mental states do modify and even cure disease. Perhaps this may account largely for differences in treatment of similar diseases or differences in medical practice, leaving out entirely the so-called "faith-cure." We know that there are a great many people who are always afflicted with some imaginary

[1] Read before the meeting of the Genesee Valley Veterinary Medical Association, January 12, 1899.

disease, and who are continually dosing themselves with medicine that they have seen advertised or that has been recommended by friends. The success of hundreds of patent nostrums is thus robbed of all mystery. Let the mind be fully convinced of the efficacy of any remedy, and that remedy may be effectual in working a complete cure. On the other hand, if the mind is alarmed or excited over the prevalence of some epidemic, the tendency will be to increase the number of cases. Thus much harm is often done by the sensational publicity given to the presence of infectious or contagious diseases in a community.

Some of you will, perhaps, remember the gentleman who came to Rochester a little over a year ago and advertised himself as the great modern healer under the name of "Derr Fritz," which means "That Fred." Possibly he was ashamed to give his right name. While we have many eminent and distinguished physicians in this city, people flocked from all directions to consult him upon their ailments, real or imaginary. So great were the crowds that many were disappointed in their hopes of an audience with him. The poor, the ignorant, the superstitious, the wealthy, the educated, and the refined fell an easy prey to this modern healer, who, while he probably had no influence over their minds and less curative power over their diseases, certainly found the way to their pocketbooks very easy.

We all know people who carry horse chestnuts in their pockets as a preventive of rheumatism. The fact is as much an "old chestnut" as the remedy itself. The anti-rheumatic ring is worn by intelligent and educated people as a safeguard against the same disease. While it probably has no more virtue than a common horseshoe-nail bent in the form of a ring, and while the wearer may declare to you that he has no faith in its powers, he will almost surely add that he has never been troubled with rheumatism since he began to wear it. The magic of the charm is governed by such influence as it may have upon the mind.

The relations between mother and offspring, both before and after birth, are so well established as to call for but little comment. The case is recorded by the London *Lancet* of a child who was playing at an open window, when the sash fell upon the index-finger of the right hand, bruising it badly and causing considerable pain and swelling. The mother, who was very much frightened, relieved the child from its painful position, and the following day, without any assignable cause, the index-finger of her right hand commenced to pain her and became badly swollen. The attending physician had no hesitancy in attributing the cause to the sympathetic influence existing between mother and child.

Of all the diseases that draw upon the imagination, perhaps none exerts a stronger influence upon the mind than the dreaded hydrophobia. The bite of a healthy dog has been known to cause symptoms of rabies in persons of excitable temperament. It is a popular belief that if a healthy dog bites a person the animal must be destroyed to prevent the one bitten from becoming affected with the disease. What satisfaction can this give or what benefit can be derived except from the effect upon the imagination? Rabies in the dog is of rare occurrence, but we often see in the newspapers a paragraph relating how some one on the street set up the cry of "mad dog," and how a policeman put a bullet through the brain of a poor brute who had no symptoms of rabies, but only a convulsive fit, which would soon pass off were the animal left alone. I am very doubtful if any one of you, gentlemen, has ever seen a genuine case of rabies. I copy here from the *Iowa Medical Journal* a statement of the case of a young married woman who manifested symptoms of hydrophobia after the loss of her only child. The death of the little one was due to convulsions caused by its being frightened by a large Newfoundland dog which jumped upon the child in a playful mood without inflicting the slightest bite or scratch. "Shortly after the death of the child the mother was taken with pains in the head, without febrile symptoms. The bowels were constipated, the urine pale, and an attempt at swallowing was undertaken with reluctance and performed with difficulty. Shortly after she became subject to paroxysms, during which she complained of thirst and demanded water, but on bringing it to her lips violent spasm of the larynx was excited. When first attempting to drink, the appearance of the water seemed to produce a slight shivering, as of dread, and after some hesitation she would hurriedly, apparently with a feeling of desperation, seize the cup, and with a sudden effort attempt to gulp it down, during which respiration was suspended. And after the effort of swallowing her conduct resembled that of an individual who had been immersed in water of a low temperature. Then followed a wild expiratory screech. During these fits her countenance manifested the wildest frenzy, and all the muscles of the body seemed tensely contracted. These paroxysms were excited by the unexpected entrance of strangers, a sudden noise, or a current of cold air." Whether these symptoms were those of hydrophobia and what produced those symptoms, I leave for you, gentlemen, to judge.

Among the papers of the late Prof. Dick a case is recorded of a farmer who was bitten by a cat : A child had also been bitten by the same animal. In the farmer symptoms of hydrophobia were developed, while the child escaped. It was stated that the man before he became affected had no dread of the disease. It came out, however, in the course of conversation with the medical attendant that his patient had a horror and dread of hydrophobia. The escape of the child is considered by the writer a strong point of argument in favor of the influence of the mind on disease, as the child had not arrived at an age when it was capable of reflection, and was not, therefore, subject to such influence.

We will now give our attention very briefly to the veterinarian and the practice of veterinary medicine. We who follow this calling cannot draw upon the imagination of our patients, as their actions are governed wholly by instinct. This we naturally consider a drawback, yet the most successful veterinarian is frequently the one who shrewdly operates upon the mind of the owner or groom, who almost always attributes some imaginary disease to the animal. There are very few who ever drew a pair of lines over a horse but pretend to know all about the diseases the animal is heir to.

The veterinarian must be a very close observer, as his professional duties bring him in contact with dumb animals who cannot indicate to him the seat of their disease or the location of their aches and pains. The veterinarian must be guided wholly by symptoms. The pulse, with its variations, is an index. The clinical thermometer is also a reliable guide. The respiratory movements should not be overlooked; the slow, quick, difficult, irregular or laborious breathing—each has its own indications. Auscultation and percussion also assist us in locating internal disease, while the general appearance of the visible mucous membrane is of great value to the veterinarian in his diagnosis.

While all of these are beneficial in locating diseased conditions, the veterinarian must be a close and keen observer of the most minute symptoms—many of which he cannot always himself describe. The appearance of the eye, the contracted or distended condition of the nostrils, the drawn-up muscles of the face, the movements of the intercostal muscles, the movements of the muscles of the flank and the drawn-up appearance of the same, the contraction of the muscles of the whole body and their general appearance—all these must be watched and studied. We have one advantage over the medical profession in that it is not always convenient, nor would it be practical, to make the same observations of general appearance in a human subject. The veterinarian, who is a practical student of the domestic animals in their normal condition, is the closest observer of minute symptoms of diseased conditions. In our profession this is a necessity, as our patients cannot make their wants intelligible or describe the nature of their complaints. For this reason I believe that the average veterinarian of to-day is a more careful observer of symptoms in the diagnosis of disease than the majority of physicians.

A good story is told of Dr. Schweninger, physician to the late Prince Bismarck. "For years the Chancellor had been troubled with facial neuralgia, and after several specialists had tried in vain to cure him, Count Herbert Bismarck suggested that Dr. Schweninger be consulted. His father assented, and the doctor came and straightway began to question and examine his patient. Unaccustomed to restraint of any kind, Bismarck could not contain himself under this ordeal, and exclaimed impatiently, 'Oh, have done with your long-winded questions. I don't see their use, and they are tiring me to death.' 'They tire you, eh?' said Schweninger, coolly. 'So you want me to cure you, and yet you won't take the trouble to answer my questions? It seems to me that you ought to have called in a veterinary surgeon, for these gentlemen cure their patients without asking them any questions.'"

One of the disadvantages under which we labor is, as I have said before, the inability of the brute creation to make it known to their attendants that they are sick. Some minor symptom is, consequently, overlooked, and the disease makes considerable progress before a veterinarian is called. The case is diagnosed and a plan of treatment decided upon. If the case is one of stomach or bowel trouble, where the animal is suffering excruciating pain, something must be done at once to produce relief, as the animal in its agony is likely to cause serious injury to himself if not brought under control. As we cannot appeal to its imagination, and as an infinitesimal dose of medicine will not have the desired effect, remedies should be given in quantities large enough to cause a marked physiological change. This is why homœopathy has proved unsuccessful in the treatment of our domestic animals.

The question is often put to the veterinarian, "Why cannot you reduce a fracture of the bones the same as the human physician?" He can; but will it pay except in the most common fracture? Will it be practical? In the first place, our patients are hard to control under such conditions. Next, it is a matter of dollars and cents to the owner. While the human physician may reduce a compound or comminuted fracture, as there is a life to be saved, his patient may be lame for life. This would not be considered a practical or profitable result in veterinary practice unless the animal is very valuable, the question being one of money. Take the case of a horse which has suffered a fracture of one of the bones of the leg. The fracture is reduced, and the animal gets well, but he is still a little lame and liable to so remain. Of what value is he to his owner? In veterinary practice he must come out from the treatment as sound as before, else we have done no good. In human practice the value of soundness would be an after-consideration.

Despite the prominence of the question of market value, I do not hesitate to say that the veterinarian is fully as likely to become interested in his patient and as anxious for his recovery as a medical physician in treating a human subject.

I am sorry to say that the decrease in prices paid for horses in the last few years had a telling effect upon the business of the veterinary surgeon. In the United States in 1897 the number of horses was 14,364,667, valued at $452,649,396—a decrease from 1883, when we had 16,206,802, valued at $992,225,185.

And yet new fields of labor are opening up for the veterinarian. Sanitary science, meat-inspection, milk-inspection, and the prevention of diseases which can be communicated from the lower animals to man—all these are within the scope of the veterinary profession, and the day is not far distant, either, when no board of health will be complete without a qualified veterinarian as one of its members.

